# Metabolomic fingerprint of coronary blood in STEMI patients depends on the ischemic time and inflammatory state

**DOI:** 10.1038/s41598-018-36415-y

**Published:** 2019-01-22

**Authors:** Martino Deidda, Cristina Piras, Giulio Binaghi, Damiana Congia, Alessandro Pani, Alberto Boi, Francesco Sanna, Angelica Rossi, Bruno Loi, Christian Cadeddu Dessalvi, Luigi Atzori, Maurizio Porcu, Giuseppe Mercuro

**Affiliations:** 1 Department of Medical Sciences and Public Health, University of Cagliari, Cagliari, Italy; 2 Department of Biomedical Sciences, University of Cagliari, Cagliari, Italy; 3 Department of Cardiology, G. Brotzu Hospital, Cagliari, Italy; 4 Catheterization Lab, G. Brotzu Hospital, Cagliari, Italy

## Abstract

In this study we investigated whether the metabolomic analysis could identify a specific fingerprint of coronary blood collected during primary PCI in STEMI patients. Fifteen samples was subjected to metabolomic analysis. Subsequently, the study population was divided into two groups according to the peripheral blood neutrophil-to-lymphocyte ratio (NLR), a marker of the systemic inflammatory response. Regression analysis was then applied separately to the two NLR groups. A partial least square (PLS) regression identified the most significant involved metabolites and the PLS-class analysis revealed a significant correlation between the metabolic profile and the total ischemic time only in patients with an NLR > 5.77.

## Introduction

Worldwide, coronary artery disease (CAD) is the single most frequent cause of death. In particular, the in-hospital mortality of ST-elevation myocardial infarction (STEMI) patients in European Society of Cardiology (ESC) countries varies between 6% and 14%, and the 6-month mortality remains at approximately 12%, with higher mortality rates in high-risk patients^1^.

Endothelial dysfunction and inflammation play a major role in the pathophysiology of acute coronary syndromes. Although their roles are not completely defined, they are especially important in plaque rupture, plaque erosion, and subsequent thrombus formation^2^. The endothelium is known to be involved in the modulation of vasoactive substances and platelet aggregation. For its part, inflammation can alter the function of endothelial cells, acting as a modulator of atherosclerotic risk factors in the impairment of arterial biology^3^. In this regard, the neutrophil-to-lymphocyte ratio (NLR) has recently been validated as a reliable inflammatory marker of atherosclerosis and as a predictor of clinical outcome in patients with various cardiovascular diseases^4^.

Furthermore, over the last ten years the use of targeted and non-targeted metabolomic approaches has allowed the identification of specific metabolic profiles for several cardiovascular diseases^5–7^, including CAD^8^. However, a limited number of studies have been conducted in patients with an STEMI^9,10^, and none have performed an analysis of coronary artery blood.

In this study, we report the preliminary findings of a metabolomic analysis performed on coronary artery blood collected during primary PCI in STEMI patients, with the aim to investigate how coronary blood fingerprint in the culprit vessels are associated with ischemic time and inflammatory state and to identify involved pathways.

## Results

### Coronary angiography

Patients were subjected to primary PCI with a median coronary ischemic time of 180 min (95% CI: 120–330 min). The culprit lesions were localized as follows: right coronary artery N = 6, left main/left anterior descending artery N = 5, and circumflex branch N = 4. Of the 15 coronary stents used, 12 were drug-eluting stents (5 with zetarolimus and 7 with everolimus) and 3 were bioresorbable vascular scaffold stents. Heparin (100 UI/kg, maximum dose 5000 UI) was administered intravenously before the procedure in all patients. The glycoprotein IIb/IIIa inhibitor abciximab was used in 14 patients.

### Metabolomics analysis

Two patients were excluded from the final analysis because of the use of a different contrast agent.

**Figure 1:**
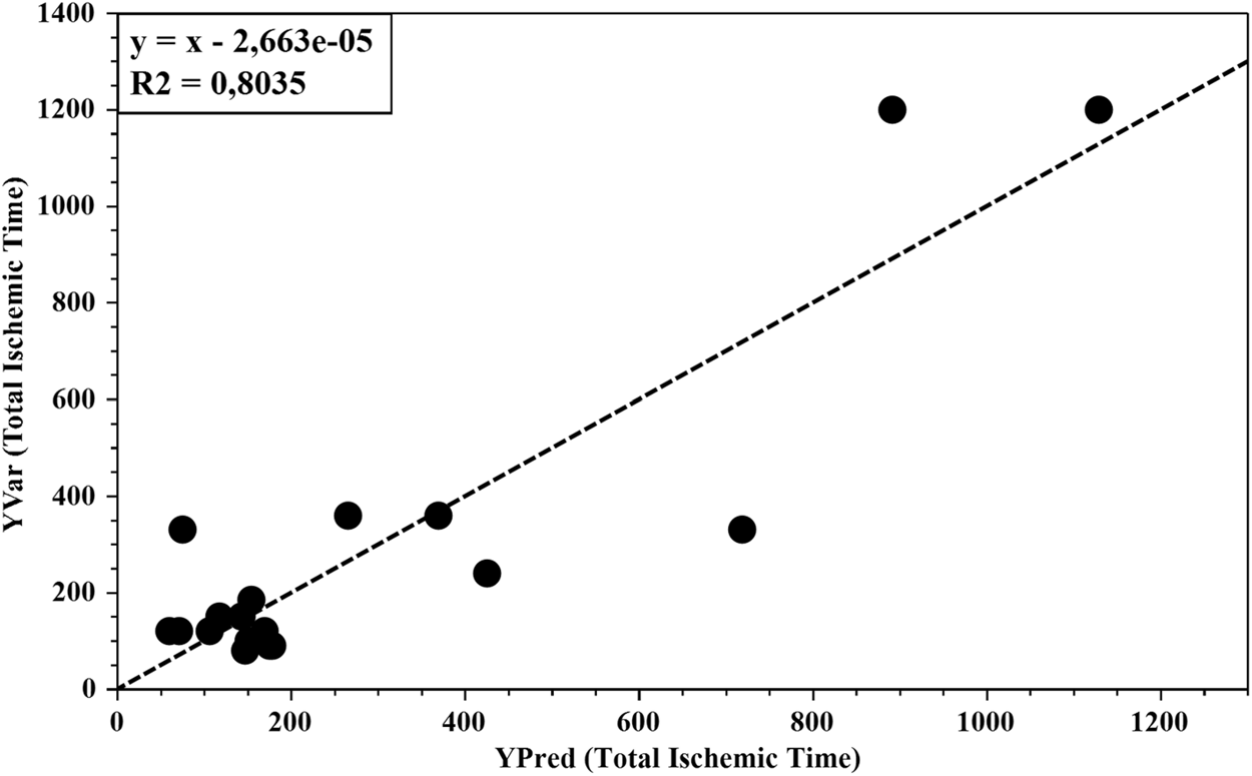
PLS plot of whole population shows the relationship between the metabolic profile and the Total Ischemic Time. The horizontal axis represents the predicted values, and the vertical axis represents the observed values.

After an unsupervised Principal Component Analysys (PCA) to visualize possible metabolic differences among the groups and to identify potential outliers, we performed a partial least square (PLS) regression (Fig. 1) using total coronary ischemic time (the time from symptom onset until reperfusion) as the Y variable, achieving a good capacity for fitting and prediction (R^2^x = 0.499; R^2^y = 0.804; Q^2^ = 0.500). PLS regression is a supervised extension of PCA (Principal Component Analysis) used to maximize the correlation between two sets of variables, e.g. spectral intensity values (X matrix) and ischemic time (Y matrix), so that the response variable Y can be predicted from X. The estimated predictive power of the model is expressed by R^2^Y and Q^2^Y, which represent the fraction of the variation of Y-variable and the predicted fraction of the variation of Y-variable, respectively. A good prediction model is achieved when Q^2^ > 0.5.

The variable interdependent parameter (VIP) analysis allowed identification of the metabolites more important in determining the culprit coronary blood fingerprint by using S-line loadings plot, which shows an increase in choline, phosphocholine, orthinitine and myo-inositol concentrations as the ischemic time increases, while lysine and 2-phosphoglycerate levels decrease.

To evaluate the effects of inflammation in modulating the endothelial response, we divided the population into two groups on the basis of the NLR, using a cut-off of 5.77^4^, into a high NLR group (N = 6) and a medium-low NLR group (N = 7). The two NLR groups showed statistically significant differences with regard to NLR (8.09 ± 1.83 vs 3.86 ± 1.62; p = 0.01) and to anthropometrics data (Height: 165 ± 4.6 cm vs 173 ± 4.86 cm, p = 0.01; Weight: 66.4 ± 11.9 Kg vs 86.6 ± 8.1 Kg, p < 0.01; BMI: Kg/m^2^ 24.1 ± 4.0 vs 28.6 ± 1.6 kg/m^2^, p < 0.01), with a negative correlation between NLR and BMI as highlighted in previous findings^11^.

**Figure 2:**
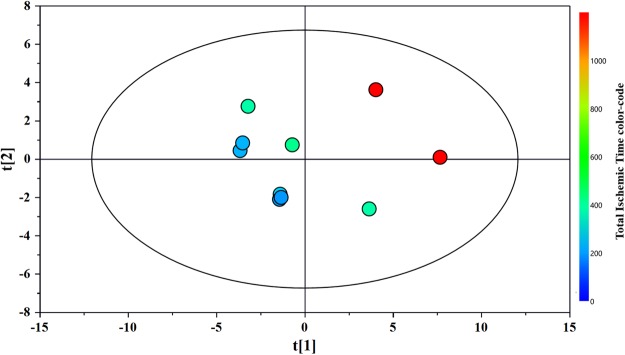
PLS score plot derived from the ^1^H-NMR spectra of patients with NLR > 5,77. PLS scores plot summarize the relationship among the observation (the samples) of the model.

Next, we applied a PLS regression analysis separately for each group. The correlation between the metabolic pool and coronary ischemic time was not significant for the group with an NLR < 5.77. In contrast, the high NLR group showed highly significant correlations between the metabolic pool and coronary ischemic time (R^2^x = 0.624; R^2^y = 0.968; Q^2^ = 0.843; Fig. 2).

## Discussion

The present study was designed to assess whether coronary blood fingerprint in the culprit vessels change on the basis of ischemic time and inflammatory state. Our results show that (1) coronary blood fingerprint in STEMI patients are constituted by choline, phosphocholine, myo-inositol, lysine, ornithine, and 2-phosphoglycerate and (2) a highly significant correlation was demonstrated between the metabolic pool and coronary ischemic time in patients with a high NLR, but not in those with an NLR < 5.77.

Alterations in endothelial function are the basic mechanism responsible for the development of atherosclerosis. These alterations play a key role in plaque progression and the development of plaque rupture, plaque erosion, and subsequent thrombus formation^2^. The metabolomic fingerprint detected in the coronary blood of our STEMI patients is characterized by a limited number of metabolites.

Choline, the immediate precursor of betaine, serves as a methyl group donor in the conversion of homocysteine to methionine and is involved in phospholipid synthesis^12^. In fact, choline is also a precursor of phosphocholine, another key metabolite in the metabolomic fingerprint, and an intermediate in the synthesis of phosphatidylcholine and of its derivates. Phosphocholine is the main water-soluble metabolite secreted by monocytes^13^ and is also the main bioactive lipid component of oxidized low density lipoprotein (LDL)^14^ and the major contributing factor in the atherogenic capacity of oxidized LDL^15^. Moreover, HDL3 subpopulations enrichment in lysophosphatidylcholine are resulted associated with deleterious biological activities during STEMI^16^.

Myo-inositol is an essential component of the plasma membrane that, when phosphorylated, acts as a second messenger. Myo-inositol levels are increased in mice fed a high cholesterol diet^17^ and in the platelets of patients with hypercholesterolemia^18^, suggesting its important role in arterial thrombosis^19^. Moreover, myo-inositol seemed to be part of the metabolic fingerprint of patients with acute coronary syndromes^20^.

Lysine is an essential amino acid that can negatively modulate the synthesis of nitric oxide (NO)^21^. In addition, ornithine is the product of the degradation of arginine by arginase. This enzyme also negatively impacts endothelial metabolism, since it degrades arginine, the precursor of NO. Higher activity of arginase II increases ornithine and reduces the production of NO, such as the inflow of lysine in endothelial cells. Higher arginase II activity has been demonstrated in the endothelial cells of patients with primary pulmonary hypertension^22^ and in the coronary endothelium of diabetic rats^23^.

2-phosphoglycerate, produced during the glycolytic process, has been shown to increase in cultured endothelial cells after the addition of LDL^24^ and is produced by activated macrophages in atherosclerotic lesions^25^, decreased as the ischemic time increases, suggesting a progressive modulation in the circulating and endothelial factors involved in the STEMI pathophysiology and a progressive impairment in energetic metabolism.

In summary, our data suggest that the systemic inflammatory state modulates the endothelial response to the occlusion, and the change of the metabolite pool becomes more intense over time. Our findings are in agreement with prior studies showing a lower patency of the infarcted artery at 60 and 90 minutes after thrombolysis in patients with neutrophilia with a relative lymphopenia^26^ and with studies showing a strong correlation between a high NLR and the phenomenon of no-reflow after primary PCI^27^. The common basis of these findings is endothelial dysfunction induced by ischemic noxa and aggravated by an imbalanced immune response, as evidenced by a high NLR.

## Conclusion

Our results indicate that (1) study of the coronary metabolomic fingerprint can allow a deeper understanding of STEMI pathophysiology, identifying involved biological pathways and (2) systemic inflammation is able to modulate the endothelial response to ischemic noxa.

### Study Limitations

The small number of samples included in this study is certainly a limitation, such as the lack of an independent validation cohort and our findings require validation in larger studies. Nevertheless, the statistical significance and the viability of the mechanisms suggested by the identified metabolites support the reliability of these preliminary results.

These preliminary data do not pretend to be exhaustive or conclusive, but want to stimulate further research in this direction, such as the relationship between metabolic disarrangement and no-reflow/slow-flow phenomenon or systolic function recovery after PCI.

## Methods

### Patients

Fifteen subjects ≥18 years of age with evidence of an STEMI according to the third universal definition of myocardial infarction^28^ were enrolled in the study.

The study was approved by the Azienda Ospedaliero-Universitaria di Cagliari Ethics Committee and was carried out in accordance with the Declaration of Helsinki and with approved guidelines, particularly all patients were treated according to the ESC STEMI guidelines^1^. Patients were informed regarding the purpose and methodology of the study, their consent was obtained and a written confirm has been collected prior to enrolment.

Exclusion criteria included previous coronary events of any type; significant (more than moderate) valvulopathy; cardiomyopathy; chronic inflammatory disease and/or cancer; and thromboembolic events or major surgery in the previous six months. Table 1 summarizes the clinical data of the study patients.

**Table 1:** Anthropometric and clinical data of the study population.

	Whole Population	NLR < 5.77	NLR > 5.77	p
	N = 13	N = 7	N = 6	
Age (years)	61.20 ± 10.92	59.4 ± 9.95	63.7 ± 11.6	0.49
Height (m)	1.68 ± 0.06	1.73 ± 4.86	1.65 ± 4.64	0.01
Weight (kg)	75.07 ± 14.52	86.5 ± 8.1	66.3 ± 11.9	<0.01
Body mass index (kg/m^2^)	26.08 ± 3.66	28.6 ± 1.57	24.1 ± 4.0	<0.01
**Ischemic time (minutes)**				
Median		120	257.5	0.17
IC 95%		80–360	120–1200	
**Cardiovascular risk factors**				
Diabetes	1	1	0	0.91
Hypertension	3	2	1	0.51
Hypercholesterolemia	4	3	1	0.23
Smoking	11	5	6	0.51

NLR: neutrophil/lymphocyte ratio.

### Blood samples during coronary angiography

All patients underwent coronary artery blood sampling near the culprit lesion using a micro-catheter inserted along a guide wire passed through the lesion. Blood samples were collected in heparinised tubes, immediately centrifuged at 4000 rpm for 15 min, aliquoted (800 μL) in cuvettes, and stored at −80 °C until metabolomics analysis.

### Metabolomics analysis

The extraction of water-soluble metabolites from plasma samples was performed on the basis of the Folch, Lees and Sloane-Stanley procedure and has been already described in previous papers of our group^29^. 400 μL of plasma were dissolved in 1.2 mL of a chloroform/methanol mixture (1:1, v/v) and 175 mL of H_2_O. The solution was finally centrifuged at 4500 rpm and 4 °C for 30 min and ~1 mL of hydrophilic phase, containing the hydrophilic components, was separated from the lipophilic one, dried using a speed vacuum concentrator (Eppendorf, Hamburg, Germany) and then stored at −80 °C. Dried hydrophilic plasma extracts were re-dissolved in 690 μL of potassium phosphate buffer in D_2_O (0.1 M, pH 7.4) and 10 μL of TSP as internal standard (98 atom % D, Sigma-Aldrich, Milan). An aliquot of 650 μL was analysed by ^1^H-NMR.

^1^H-NMR experiments were conducted using a Varian UNITY INOVA^®^ 500 spectrometer (Varian Medical Systems of Palo Alto, California, USA) operating at 499.839 MHz for protons and equipped with a 5-mm double-resonance probe (Agilent Technologies, Santa Clara, CA, USA). ^1^H-NMR spectra were acquired with a spectral width of 6000 Hz, a 90° pulse, an acquisition time of 2 s, a relaxation delay of 2 s, and 256 scans. A presaturation sequence was used to suppress the residual H_2_O signal with low power radiofrequency irradiation for 2 s. ^1^H-NMR spectra were imported into an ACDlab Processor Academic Edition (version 12.01, 2010, Advanced Chemistry Development, Toronto, Canada) and pre-processed with line broadening of 0.1 Hz, zero-filled to 64 K, prior to Fourier transformation. Spectra were manually phased and baseline corrected and chemical shifts referenced internally to TSP at δ = 0.0 ppm. The ^1^H-NMR spectra were reduced into consecutive integrated spectral regions (bins) of 0.01 ppm. Were excluded from the analysis the spectral regions between 1.87–1.99 ppm, 2.36–2.50 ppm, 3.32, 3.55 ppm, 3.68–4.14 ppm and 4.74–4.49 in order to remove the resonances of contrast agent and the effect of variation in the pre-saturation of the residual water resonance

Finally, the spectral data set was normalized to the total area and imported into the SIMCA-P + program (Version 14.0, Umetrics, Umea, Sweden). All imported data were Pareto scaled for multivariate analysis. Two ^1^H-NMR spectra are shown in the Electronic supplementary material.

### Univariate statistical analysis

Continuous variables were compared with a non-paired t-test, and categorical variables were compared with Fisher’s exact test. A two-tailed p value < 0.05 was considered statistically significant.

## Electronic supplementary material

Supplementary information
