# Supplementary material for: Metabolomic fingerprint of coronary blood in STEMI patients depends on the ischemic time and inflammatory state
Source: Sci Rep. 2019 Jan 22;9:312. doi: 10.1038/s41598-018-36415-y (PMC6342950; doi:10.1038/s41598-018-36415-y)
Supplement: Supplementary file 1 — Supplementary information [file 41598_2018_36415_MOESM1_ESM.pdf]

Metabolomic fingerprint of coronary blood in STEMI patients depends on the ischemic time and inflammatory state

[a]Martino Deidda, MD PhD; [b]Cristina Piras, PhD; [c]Giulio Binaghi, MD PhD; [c]Damiana Congia, MD; [c]Alessandro Pani, Md; [d]Alberto Boi, MD; [d]Francesco Sanna, MD; [d]Angelica Rossi, MD; [d]Bruno Loi, MD; [a]Christian Cadeddu Dessalvi, MD PhD FESC; [b]Luigi Atzori, MD; [c]Maurizio Porcu, MD FESC; [a]Giuseppe Mercuro, MD

[a] Department of Medical Sciences and Public Health, University of Cagliari; [b] Department of Biomedical Sciences, University of Cagliari; [c] Department of Cardiology, G. Brotzu Hospital; [d] Catheterization Lab, G. Brotzu Hospital, Cagliari, Sardinia, Italy

Figure 1S. 500 MHz 1H NMR spectra of plasma extracts obtained from a) short total ischemic time b) long total ischemic time.

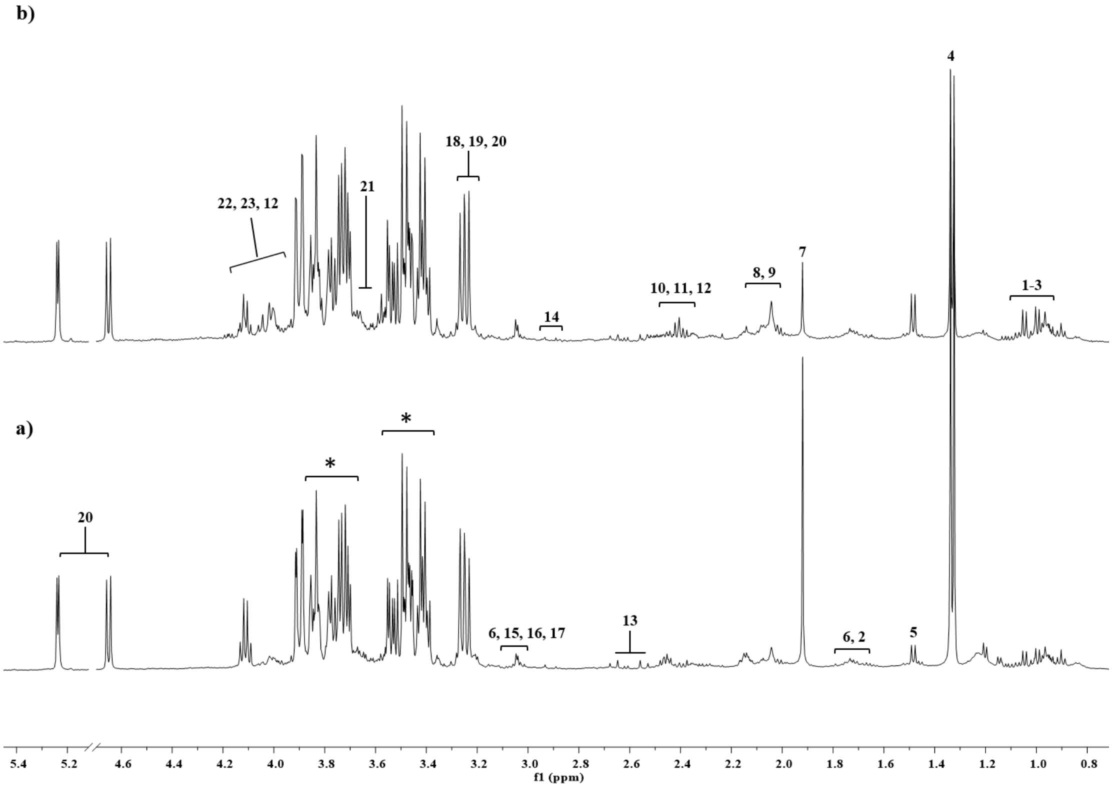

Peaks: 1, Isoleucine; 2, Leucine; 3, Valine, 4, Lactate; 5, Alanine; 6, Lysine; 7, Acetate; 8, N-acetylgroups; 9, Proline; 10, Glutamine; 11, Glutamate; 12, Pyroglutamate; 13, Citrate; 14, Asparagine; 15, Creatine; 16, Creatinine; 17, Ornithine; 18, Choline; 19, Phosphocholine; 20, Glucose; 21, Myo-Inositol; 22, 2-phosphoglycerate; 23, Serine. * Resonances of contrast agent.
